# Deucravacitinib in plaque psoriasis: Safety and efficacy through 3 years in Japanese patients in the phase 3 POETYK PSO‐1, PSO‐4, and LTE trials

**DOI:** 10.1111/1346-8138.17685

**Published:** 2025-03-11

**Authors:** Akimichi Morita, Shinichi Imafuku, Yayoi Tada, Yukari Okubo, Katsuyoshi Habiro, Katsuki Tsuritani, Subhashis Banerjee, Kim Hoyt, Renata M. Kisa, Mamitaro Ohtsuki

**Affiliations:** ^1^ Nagoya City University Graduate School of Medical Sciences Nagoya Japan; ^2^ Fukuoka University Faculty of Medicine Fukuoka Japan; ^3^ Teikyo University School of Medicine Tokyo Japan; ^4^ Tokyo Medical University Tokyo Japan; ^5^ Bristol Myers Squibb Tokyo Japan; ^6^ Bristol Myers Squibb Princeton New Jersey USA; ^7^ Jichi Medical University Tochigi Japan

**Keywords:** long‐term, phase 3, psoriasis, safety, tyrosine kinase 2 inhibitor

## Abstract

Deucravacitinib, an oral, selective, allosteric tyrosine kinase 2 inhibitor, was effective and well tolerated at a dose of 6 mg once daily through 1 year (52 weeks) in patients with moderate to severe plaque psoriasis in the phase 3 POETYK PSO‐1 and POETYK PSO‐4 trials. Patients completing PSO‐1 or PSO‐4 could enter the ongoing POETYK long‐term extension trial and receive open‐label deucravacitinib. Safety and efficacy were evaluated through 3 years (148 weeks; data cutoff date: June 15, 2022) in Japanese patients in these trials. Safety was assessed via adverse events (AEs). Efficacy endpoints, including ≥75% reduction from baseline in the Psoriasis Area and Severity Index (PASI 75) and static Physician Global Assessment (sPGA) score of 0/1 (clear/almost clear), were evaluated in patients receiving continuous deucravacitinib treatment from baseline in PSO‐1 and PSO‐4 and in PSO‐1 patients crossing over from placebo to deucravacitinib at week 16. At data cutoff, 125 patients had received at least one deucravacitinib dose; 86.4% had >24 months and 27.2% had >36 months of total deucravacitinib exposure. Exposure‐adjusted incidence rates per 100 person‐years for AEs were: any AEs, 188.5; discontinuations attributable to AEs, 3.2; serious AEs, 7.4; serious infections, 1.3; herpes zoster events, 1.6; major adverse cardiovascular events, 0.6; venous thromboembolic events, 0; and malignancies, 1.0. Clinical responses (as observed) were maintained in PSO‐1 patients receiving continuous deucravacitinib treatment from baseline (PASI 75: year 1, 88.9%; year 3, 87.5%; sPGA 0/1: year 1, 74.1%; year 3, 66.7%). Year 1 response rates were also maintained through year 3 in PSO‐4 patients and in PSO‐1 placebo crossovers. Response rates were also consistent using modified nonresponder imputation and treatment failure rules data imputation methodologies. These findings support the consistent safety profile and durable efficacy of deucravacitinib through 3 years in Japanese patients with psoriasis.

ClinicalTrials.gov: NCT03624127; NCT03924427; NCT04036435.

## 1 INTRODUCTION

Plaque psoriasis is a chronic, immune‐mediated inflammatory disease that may impair physical and mental health and reduce work productivity and quality of life.^1^ Globally, psoriasis affects more than 125 million people, and the incidence is increasing.^1^ The prevalence of psoriasis in Japan is 0.34% (95% confidence interval, 0.34%–0.34%).^2^ In a retrospective study of systemic therapy use in Japanese patients with plaque psoriasis, the mean time between initial plaque psoriasis diagnosis and starting a systemic drug was 7.8 years, and most patients began with an oral systemic therapy.^3^ However, for many patients, these oral drugs were less effective and/or were associated with safety concerns, which led to more frequent treatment changes.^3^ Although currently available biologics and oral small‐molecule agents have improved patient outcomes in psoriasis,^1^, ^4^ there remains an unmet need for a treatment that can provide long‐term maintenance of efficacy, address safety and tolerability concerns, and offer convenient dosing.^5^, ^6^ New treatments with improved risk–benefit profiles based on selective targeting of specific signaling pathways involved in psoriasis are also needed.^1^, ^4^

Tyrosine kinase 2 (TYK2) is an intracellular enzyme that mediates cytokine signaling (e.g., interleukin [IL] 23, IL‐12, type I interferons); IL‐23 and type I interferons are involved in psoriasis pathogenesis.^7^, ^8^ Deucravacitinib, an oral, selective, allosteric TYK2 inhibitor, uniquely binds to the less conserved regulatory (pseudokinase) domain of TYK2 rather than to the more conserved catalytic domain where Janus kinase (JAK) type 1, 2, and 3 inhibitors bind,^7^, ^9^ driving its selectivity and representing the first in a new class of oral drugs. Deucravacitinib is approved in the United States, European Union, Japan, South Korea, China, and other countries for the treatment of moderate to severe plaque psoriasis in adults who are candidates for systemic therapy.^10^, ^11^, ^12^, ^13^, ^14^, ^15^, ^16^ Deucravacitinib is also approved in Japan for generalized pustular psoriasis or erythrodermic psoriasis in patients who have had an inadequate response to conventional therapies.^11^

Two global, phase 3, double‐blind, placebo‐ and apremilast‐controlled trials, POETYK PSO‐1 (NCT03624127) and POETYK PSO‐2 (NCT03611751), demonstrated that deucravacitinib was significantly more effective than placebo (based on the coprimary endpoints of ≥75% reduction from baseline in Psoriasis Area and Severity Index [PASI 75] and static Physician Global Assessment [sPGA] score of 0 (clear) or 1 (almost clear) with a ≥2‐point improvement from baseline [0/1] at week 16) and apremilast and was well tolerated in patients with moderate to severe plaque psoriasis.^17^, ^18^ Clinical responses were maintained through 1 year (52 weeks) in patients receiving continuous deucravacitinib treatment from baseline.^17^, ^18^ In a subanalysis of Japanese patients with moderate to severe plaque psoriasis in PSO‐1 (PSO‐2 did not include Japanese patients), deucravacitinib demonstrated numerically higher efficacy response rates versus placebo at week 16 and versus apremilast at week 24, and was well tolerated.^19^ Response rates were maintained through 1 year in these Japanese patients receiving continuous deucravacitinib treatment from baseline.^19^ In a phase 3, regional, open‐label trial in Japanese patients with moderate to severe plaque psoriasis, generalized pustular psoriasis, or erythrodermic psoriasis (POETYK PSO‐4; NCT03924427), deucravacitinib was also effective and well tolerated through 1 year.^20^ Safety and efficacy outcomes through 1 year in the Japanese patient subgroup in PSO‐1 and in the study population in PSO‐4 were consistent with those reported in the global study populations in PSO‐1 and PSO‐2.^17^, ^18^, ^19^, ^20^ Patients completing PSO‐1, PSO‐2, and PSO‐4 could enroll in the POETYK long‐term extension (LTE) (NCT04036435) trial and receive open‐label deucravacitinib treatment.^21^ As previously reported, deucravacitinib‐treated patients in the overall PSO‐1 and PSO‐2 study populations maintained long‐term efficacy responses through 3 years (148 weeks) with no new safety signals observed compared with those reported through 1 year or 2 years.^21^, ^22^ The objective of the current analysis was to evaluate the safety and efficacy of long‐term deucravacitinib treatment through 3 years in Japanese patients with moderate to severe plaque psoriasis in PSO‐1 and PSO‐4.

## 2 METHODS

### 2.1 Study designs and patients

**FIGURE 1:**
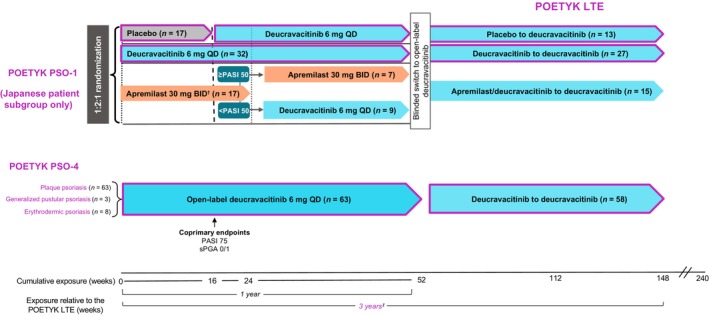
The designs of the POETYK PSO‐1, PSO‐4, and long‐term extension (LTE) studies, which include Japanese patients who received at least one dose of deucravacitinib 6 mg once daily (QD). The study design of PSO‐1 in the global population has been previously described.^17 †^Apremilast was titrated from 10 mg QD to 30 mg twice daily (BID) during the first 5 days of dosing. ^‡^Data reported through 3 years (data cutoff date: June 15, 2022). PASI 50/75, ≥50%/≥75% reduction from baseline in Psoriasis Area and Severity Index; sPGA 0/1, static Physician Global Assessment score of 0 (clear) or 1 (almost clear) with a ≥2‐point improvement from baseline.

The PSO‐1, PSO‐4, and LTE study designs have been previously described.^19^, ^20^, ^21^ Briefly, PSO‐1 was a global, 1‐year, phase 3, double‐blind trial that randomized adults (≥18 years of age) with stable moderate to severe plaque psoriasis (defined as PASI ≥12, sPGA ≥3, and body surface area involvement ≥10% for ≥6 months) 1:2:1 to oral placebo, deucravacitinib 6 mg once daily, or apremilast 30 mg twice daily (Figure 1).^19^ Patients randomized to placebo crossed over to deucravacitinib at week 16, patients randomized to deucravacitinib continued the same treatment through 1 year, and patients randomized to apremilast who did not achieve ≥50% reduction from baseline in PASI (PASI 50) switched to deucravacitinib at week 24. The subgroup of Japanese patients in PSO‐1 was analyzed in this report. POETYK PSO‐4 was a 1‐year, phase 3, single‐arm trial that included Japanese adults (≥20 years of age) with moderate to severe plaque psoriasis, moderate to severe generalized pustular psoriasis (erythematous pustules with ≥10% body surface area involvement, skin lesion score ≥2 at screening and day 1, total Japanese Dermatological Association severity index score <14 at screening, receiving a stable treatment regimen for ≥2 weeks before day 1), or moderate to severe erythrodermic psoriasis (≥80% body surface area involvement at screening and day 1, history of plaque psoriasis). All patients received open‐label deucravacitinib 6 mg once daily for 1 year (Figure 1).^20^ At week 52, patients in PSO‐1 and PSO‐4 were eligible to enroll in the LTE trial and receive open‐label deucravacitinib 6 mg once daily.

Clinical trials were conducted in accordance with Good Clinical Practice, as defined by the International Council for Harmonization and the Declaration of Helsinki. Each clinical trial protocol was approved by an institutional review board or independent ethics committee at the study site, and all patients provided written informed consent before any study‐related procedures were performed.

### 2.2 Safety and efficacy analysis populations

Safety was assessed in the as‐treated population (i.e., patients receiving at least one dose of deucravacitinib) through 3 years (data cutoff date: June 15, 2022) in Japanese patients in the pooled PSO‐1, PSO‐4, and LTE populations. Efficacy was assessed through 3 years (week 148) separately in Japanese PSO‐1 and PSO‐4 patients who had entered the LTE trial. Japanese patients in PSO‐1 who had received continuous deucravacitinib treatment from baseline (day 1) of the parent trial through 3 years and those who crossed over from placebo at week 16 and received deucravacitinib treatment through 3 years were included. Patients with plaque psoriasis in PSO‐4 who had received continuous deucravacitinib treatment from day 1 through 3 years were evaluated; because of the low numbers, patients with generalized pustular psoriasis (*n* = 2) or erythrodermic psoriasis (*n* = 6) were not included in this analysis.

### 2.3 Safety and efficacy outcomes

The primary endpoint in the LTE trial was safety, with efficacy being a secondary endpoint.^21^ Adverse events (AEs), serious AEs, deaths, AEs leading to treatment discontinuation, and AEs of interest were evaluated using the Medical Dictionary for Regulatory Activities (MedDRA) version 25.0 in the as‐treated population through the data cutoff date. AEs of interest included select infections (serious, opportunistic, fungal, tuberculosis, herpes zoster), major adverse cardiovascular events (cardiovascular death, nonfatal myocardial infarction, nonfatal stroke), thromboembolic events (arterial and venous), malignancies, and skin events (acne and folliculitis). These events were identified based on comorbidities associated with psoriasis (eg, cardiovascular disease), the tolerability profile of deucravacitinib previously described in phase 1 and 2 trials, and AEs reported with other approved psoriasis therapies.^1^, ^23^, ^24^ Safety data were based on AEs being ascribed to the treatment group that patients were assigned to at the time of first occurrence. Standard laboratory parameters were evaluated through 3 years, with creatine phosphokinase (CPK) included in this report, as elevations in this parameter were the most common laboratory abnormality reported as an AE across the three treatment groups in the global POETYK PSO‐1 and PSO‐2 trials. CPK elevations in the global trials were associated with recent physical exertion, were transient, and usually resolved without treatment.^17^, ^18^ Efficacy outcomes through 3 years included PASI 75, ≥90% reduction from baseline in PASI (PASI 90), 100% reduction from baseline in PASI (PASI 100), and sPGA 0/1 response rates.

### 2.4 Statistical analysis

AEs are presented as frequencies and exposure‐adjusted incidence rates (EAIRs) per 100 person‐years (PYs) to account for variable durations of treatment exposure arising from treatment switches at weeks 16 and 24. CPK levels were summarized as mean values. Efficacy outcomes were summarized using the prespecified as‐observed analysis without imputation of any missing data. Consistent with precedents for assessing long‐term outcomes in psoriasis clinical trials, sensitivity analyses of efficacy outcomes were performed using two methods to impute missing data.^25^ The first method was modified nonresponder imputation (mNRI), which assessed missing values for patients who either discontinued prior to 3 years or reached 3 years; those with missing data who discontinued treatment because of worsening of psoriasis were imputed as nonresponders. All other missing data were imputed by multiple imputation.^26^ The second method was treatment failure rules (TFRs), which imputed missing values as nonresponses only for patients who discontinued treatment because of lack of efficacy or worsening of psoriasis; all other missing data were not imputed.^27^ The 95% confidence intervals were determined using the Clopper‐Pearson method. Given that this was a subgroup analysis of PSO‐1 and an open‐label analysis of PSO‐4, no formal statistical tests were performed to evaluate safety and efficacy outcomes with long‐term deucravacitinib treatment.

## 3 RESULTS

### 3.1 Patient population and treatment exposure

At data cutoff (week 148), 125 Japanese patients had received at least one dose of deucravacitinib through 3 years in the combined PSO‐1/LTE (*n* = 62) and PSO‐4/LTE (*n* = 63) trials. A total of 86.4% of patients had >24 months and 27.2% had >36 months of total deucravacitinib exposure (Table 1). The median duration of exposure was 1036.0 days (range, 8.0–1303.0 days) and total exposure was 322.7 PYs through 3 years.

**TABLE 1:** Extent of exposure in Japanese patients with plaque psoriasis in the pooled POETYK PSO‐1, PSO‐4, and LTE populations.

Exposure	Deucravacitinib^a^ (*n* = 125)
≥16 weeks (≥4 months) of exposure, *n* (%)	121 (96.8)
>12 months of exposure, *n* (%)	112 (89.6)
>24 months of exposure, *n* (%)	108 (86.4)
>36 months of exposure, *n* (%)	34 (27.2)
Total exposure, PYs	322.7
Median (range) exposure, days	1036.0 (8–1303)

Abbreviations: LTE, long‐term extension; PY, person‐year.

^a^ Pooled Japanese patients from the parent trials (POETYK PSO‐1 and PSO‐4) and the POETYK LTE who received ≥1 dose of deucravacitinib through the data cutoff date of June 15, 2022, were included.

Baseline demographics and disease characteristics were typical of patients with moderate to severe plaque psoriasis; most patients were men (76.0%), with a mean age of 48.8 years (standard deviation [SD], 11.2 years), mean body mass index of 25.3 kg/m^2^ (SD, 4.7 kg/m^2^), and mean disease duration of 14.1 years (SD, 9.5 years) (Table 2). Of the 125 patients included at the data cutoff of 3 years, 102 (81.6%) were receiving ongoing deucravacitinib treatment, three (2.4%) had completed treatment through 1 year in PSO‐1 and PSO‐4 but did not enter the LTE trial, and 20 (16.0%) had discontinued treatment (9 [7.2%] during 1 year in the PSO‐1 [deucravacitinib, placebo, and apremilast arms] and PSO‐4 parent trials; 11 [8.8%] in the LTE trial). The most common reasons for treatment discontinuation were AEs, other reasons, noncompliance with the study protocol, and patient withdrawal (Figure S1).

**TABLE 2:** Baseline demographics and disease characteristics of Japanese patients with plaque psoriasis in the pooled POETYK PSO‐1, PSO‐4, and LTE populations.

Parameter	Deucravacitinib^a^ (*n* = 125)
Age, mean (SD), years	48.8 (11.2)
Weight, mean (SD), kg	71.0 (15.2)
Body mass index, mean (SD), kg/m^2^	25.3 (4.7)
Men, *n* (%)	95 (76.0)
Age at disease onset, mean (SD), years	35.6 (12.7)
Disease duration, mean (SD), years	14.1 (9.5)
PASI, mean (SD)	21.6 (8.6)
sPGA score, *n* (%)	
3 (moderate)	109 (87.2)
4 (severe)	16 (12.8)
Body surface area involvement, mean (SD), %	29.3 (16.8)
Prior systemic therapy use, *n* (%)	82 (65.6)
Nonbiologic	56 (44.8)
Biologic	26 (20.8)
No prior systemic therapy use, *n* (%)	43 (34.4)

Abbreviations: LTE, long‐term extension; PASI, Psoriasis Area and Severity Index; SD, standard deviation; sPGA, static Physician Global Assessment.

^a^ Pooled Japanese patients from the parent trials (POETYK PSO‐1 and PSO‐4) and the POETYK LTE who received ≥1 dose of deucravacitinib through the data cutoff date of June 15, 2022, were included.

### 3.2 Safety

The EAIRs of AEs through 3 years in the combined Japanese population from the two trials were 188.5/100 PYs, serious AEs were 7.4/100 PYs, and AEs resulting in treatment discontinuation were 3.2/100 PYs (Table 3; Table S1). The most frequently reported AEs (EAIR ≥5/100 PYs) through 3 years were nasopharyngitis (EAIR, 20.0/100 PYs) and psoriasis (EAIR, 5.6/100 PYs). No deaths occurred during the 3‐year period.

**TABLE 3:** Cumulative safety summary and AEs of interest through 3 years in Japanese patients with plaque psoriasis in the pooled POETYK PSO‐1, PSO‐4, and LTE (as‐treated) populations.

AE category	Deucravacitinib^a^ (*n* = 125)	
	Total exposure, PYs = 322.7	
	*n* (%)	EAIR/100 PYs (95% CI)
AEs	118 (94.4)	188.5 (157.3–225.7)
Serious AEs	21 (16.8)	7.4 (4.8–11.4)
Deaths	0	0
Discontinued treatment because of AEs^b^	10 (8.0)	3.2 (1.7–6.0)
Most common AEs (EAIR/100 PYs ≥5)		
Nasopharyngitis	45 (36.0)	20.0 (14.9–26.8)
Psoriasis	16 (12.8)	5.6 (3.4–9.1)
AEs of interest		
Serious infections	4 (3.2)	1.3 (0.5–3.5)
COVID‐19	2 (1.6)	0.6 (0.2–2.6)
Pneumonia	1 (0.8)	0.3 (0.0–2.3)
Pyelonephritis	1 (0.8)	0.3 (0.0–2.3)
Infections of interest		
Herpes zoster	5 (4.0)	1.6 (0.7–3.9)
Influenza	4 (3.2)	1.3 (0.5–3.5)
COVID‐19	3 (2.4)	1.0 (0.3–3.0)
Opportunistic	0	0
Tuberculosis	0	0
Hepatitis B	0	0
Cardiovascular		
MACE^c^	2 (1.6)	0.6 (0.2–2.6)
Acute myocardial infarction^d^	1 (0.8)	0.3 (0.0–2.3)
Cerebral infarction^e^	1 (0.8)	0.3 (0.0–2.3)
Unstable angina	2 (1.6)	0.6 (0.2–2.6)
Angina pectoris	1 (0.8)	0.3 (0.0–2.3)
ATE and VTE	0	0
Malignancies	3 (2.4)	1.0 (0.3–3.0)
Colorectal cancer	1 (0.8)	0.3 (0.0–2.3)
Gastric cancer	1 (0.8)	0.3 (0.0–2.3)
Acute promyelocytic leukemia	1 (0.8)	0.3 (0.0–2.3)
Skin events of interest (EAIR/100 PYs, ≥2)	49 (39.2)	22.2 (16.7–29.3)
Acneiform rash	13 (10.4)	4.5 (2.6–7.8)
Acne	9 (7.2)	3.1 (1.6–5.9)
Dermatitis, contact	8 (6.4)	2.7 (1.3–5.3)
Folliculitis	8 (6.4)	2.6 (1.3–5.3)
Urticaria	7 (5.6)	2.4 (1.1–4.9)
Eczema	6 (4.8)	2.0 (0.9–4.4)
Rhabdomyolysis	0	0
Blood CPK increased	5 (4.0)	1.7 (0.7–4.0)

Abbreviations: AE, adverse event; ATE, arterial thromboembolic event; CI, confidence interval; CPK, creatine phosphokinase; EAIR, exposure‐adjusted incidence rate; LTE, long‐term extension; MACE, major adverse cardiovascular event; PY, person‐year; VTE, venous thromboembolic event.

^a^ Pooled Japanese patients from the parent trials (POETYK PSO‐1 and PSO‐4) and the POETYK LTE who received ≥1 dose of deucravacitinib through the data cutoff date of June 15, 2022, were included.

^b^ Patients discontinued treatment because of malignancies (acute promyelocytic leukemia, colorectal cancer, and gastric cancer; *n* = 1 each), acne, alcoholism, chronic inflammatory demyelinating polyradiculoneuropathy, folliculitis, hepatic function abnormal, neutrophil count decreased, and psoriasis (*n* = 1 each).

^c^ MACE is defined as cardiovascular death, nonfatal myocardial infarction, and nonfatal stroke.

^d^ A 68‐year‐old man with a history of heart enlargement, hypertension, hyperlipidemia, alcohol use, and smoking was hospitalized for life‐threatening acute myocardial infarction 2 years and 7 months after initiating deucravacitinib treatment for psoriasis. Cardiac catheterization revealed 90% stenosis of the right coronary artery and total occlusion of the left circumflex branch. Percutaneous transluminal angioplasty was performed and successfully resolved the acute myocardial infarction, which was assessed as unrelated to treatment by the investigator. Deucravacitinib treatment was interrupted during the event and was subsequently resumed, with the patient remaining in the POETYK LTE trial.

^e^ A 50‐year‐old woman with a history of hypertension, diabetes, obesity, and smoking had an occurrence of cerebral infarction 3 months after initiating deucravacitinib treatment for psoriasis. After the patient received appropriate treatment for the cerebral infarction, the event resolved. The cerebral infarction was assessed by the investigator as unrelated to treatment. Deucravacitinib treatment was interrupted during the event and was subsequently resumed.

[Correction added on 18 April 2025 after first online publication: In Table 3, “Acne” has been indented.].

Incidences of AEs of interest, including serious infections, herpes zoster events, major adverse cardiovascular events, arterial thromboembolic or venous thromboembolic events, and malignancies, in the Japanese population during the 3‐year period are presented in Table 3. The EAIR of serious infections during the 3‐year period was 1.3/100 PYs; serious infections included COVID‐19 (EAIR, 0.6/100 PYs), pneumonia (EAIR, 0.3/100 PYs), and pyelonephritis (EAIR, 0.3/100 PYs); most infections resolved with appropriate therapy and did not require treatment discontinuation. No tuberculosis events, opportunistic infections, or hepatitis B reactivation events were reported. In PSO‐4, one patient tested positive for the hepatitis B core antibody at baseline; this patient continued to receive deucravacitinib treatment through 3 years in the LTE trial without hepatitis B reactivation. No herpes zoster event (*n* = 5; EAIR, 1.6/100 PYs) was serious, disseminated, or resulted in treatment discontinuation; most events resolved after treatment interruption and/or use of antiviral therapy. None of the patients who developed herpes zoster reactivation events had a history of herpes zoster or reported receiving a herpes zoster vaccination. Adjudicated major adverse cardiovascular events occurred in two patients (EAIR, 0.6/100 PYs): acute myocardial infarction was reported in one patient with a history of heart enlargement, hypertension, hyperlipidemia, alcohol use, and smoking; cerebral infarction was reported in another patient with a history of hypertension, diabetes, obesity, and smoking. Neither event was considered treatment‐related by the investigator. No venous thromboembolic events were reported during the 3‐year period. Malignancy was reported in three patients (EAIR, 1.0/100 PYs) during the 3‐year period (one each with colorectal cancer, gastric cancer, and acute promyelocytic leukemia). Skin events of interest—acne (EAIR, 3.1/100 PYs) and folliculitis (EAIR, 2.6/100 PYs)—were nonserious; one patient discontinued deucravacitinib treatment because of facial acne and another because of nose tip folliculitis during the 3‐year period.

**FIGURE 2:**
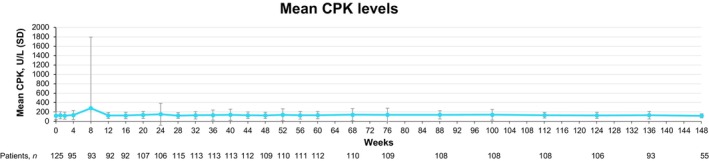
Creatine phosphokinase (CPK) levels through 3 years in Japanese patients with plaque psoriasis in the pooled POETYK PSO‐1, PSO‐4, and long‐term extension trials. In PSO‐1, a 35‐year‐old Asian man had a grade 4 CPK elevation (14 707 U/L) at week 8 and a 30‐year‐old Asian man had a grade 3 CPK elevation (2451 U/L) at week 24; both events had resolved during subsequent visits on continued treatment with deucravacitinib. All other CPK elevations were grade ≤2 (≤1283 U/L).

No clinically relevant changes were observed in mean CPK levels through 3 years (Figure 2). An AE of CPK elevation was observed in five patients (EAIR, 1.7/100 PYs) during the 3‐year period (grade 1, *n* = 1; grade 2, *n* = 2; grade 3, *n* = 1; grade 4, *n* = 1) (Table 3). Most CPK elevations were grade ≤2, transient, and resolved with continuous deucravacitinib treatment. No cases of rhabdomyolysis were reported, and no patients discontinued treatment because of CPK elevations.

### 3.3 Efficacy

**FIGURE 3:**
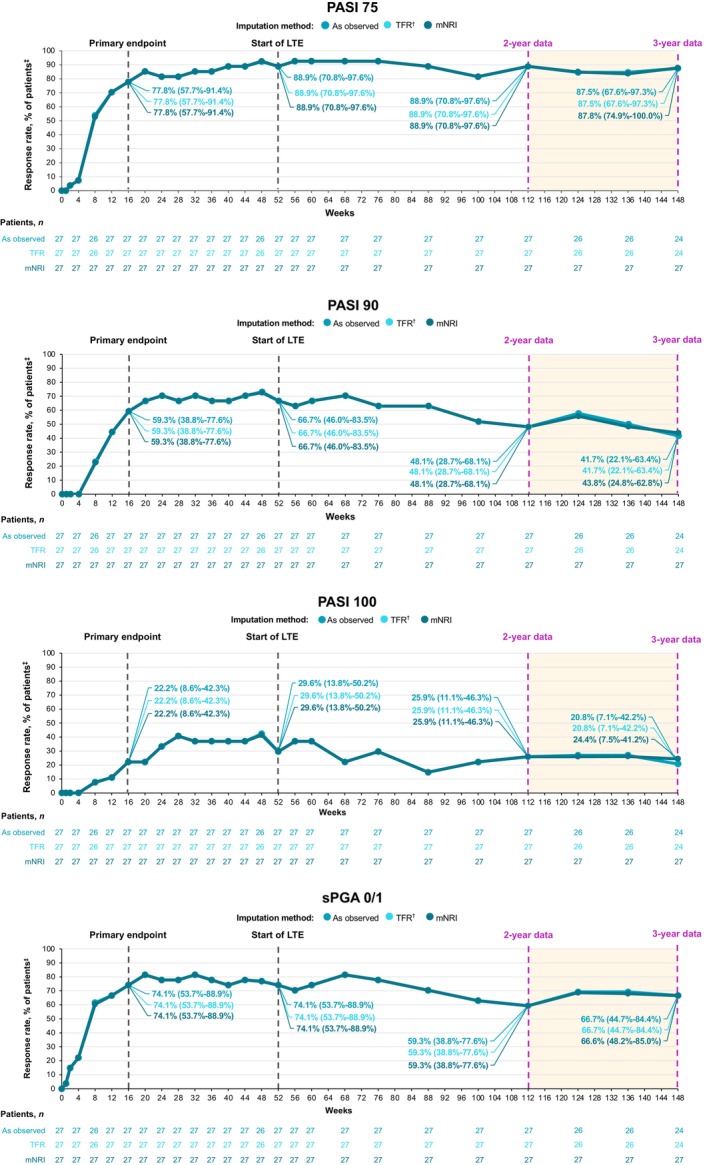
Efficacy is reported through 3 years in Japanese patients with plaque psoriasis receiving continuous deucravacitinib treatment in POETYK PSO‐1. ^†^Treatment failure rule (TFR) analysis captures discontinuations coded as “lack of efficacy” or “worsening of psoriasis.” ^‡^Modified nonresponder imputation (mNRI) analysis captures discontinuations coded as “worsening of psoriasis.” Data callouts represent the response rate (95% confidence interval]). LTE, long‐term extension; PASI 75/90/100, ≥75%/≥90%/100% reduction from baseline in Psoriasis Area and Severity Index; sPGA 0/1, static Physician Global Assessment score of 0 (clear) or 1 (almost clear) with ≥2‐point improvement from baseline.[Correction added on 18 April 2025 after first online publication: The legend of figure 3 has been modified.]

**FIGURE 4:**
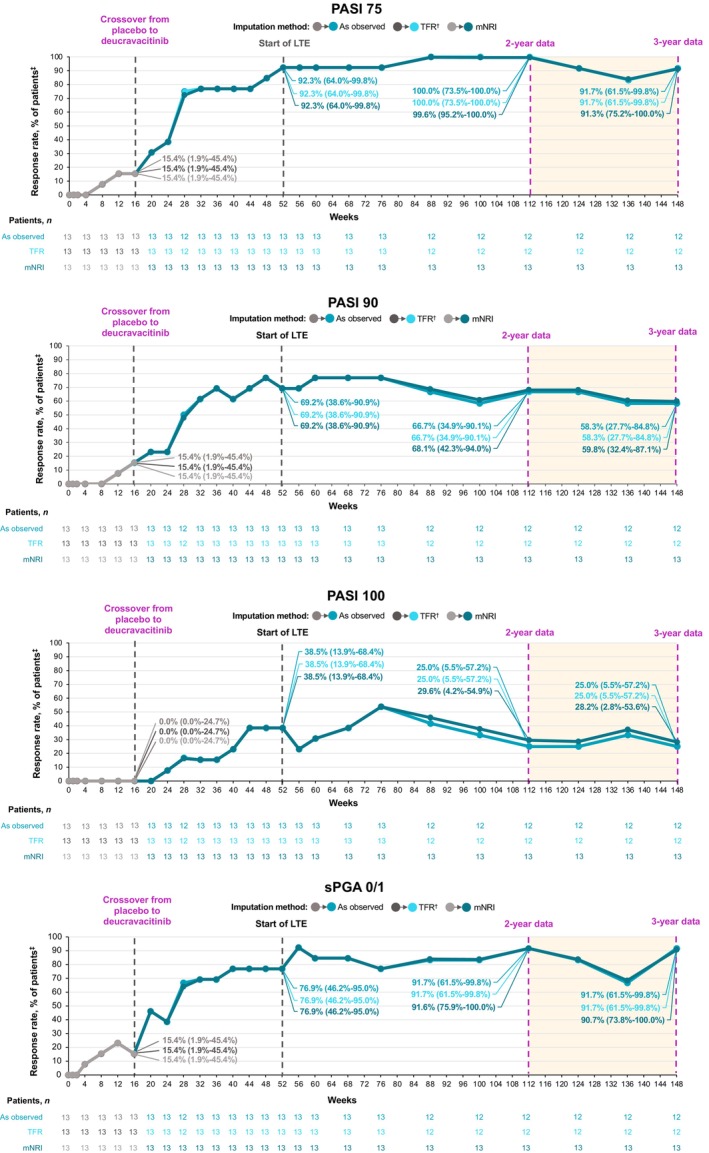
Efficacy is reported through 3 years in Japanese patients with plaque psoriasis crossing over from placebo to deucravacitinib at week 16 in POETYK PSO‐1. ^†^Treatment failure rule (TFR) analysis captures discontinuations coded as “lack of efficacy” or “worsening of psoriasis.” ^‡^Modified nonresponder imputation (mNRI) analysis captures discontinuations coded as “worsening of psoriasis.” Data callouts represent the response rate (95% confidence interval). LTE, long‐term extension; PASI 75/90/100, ≥75%/≥90%/100% reduction from baseline in Psoriasis Area and Severity Index; sPGA 0/1, static Physician Global Assessment score of 0 (clear) or 1 (almost clear) with ≥2‐point improvement from baseline.

**FIGURE 5:**
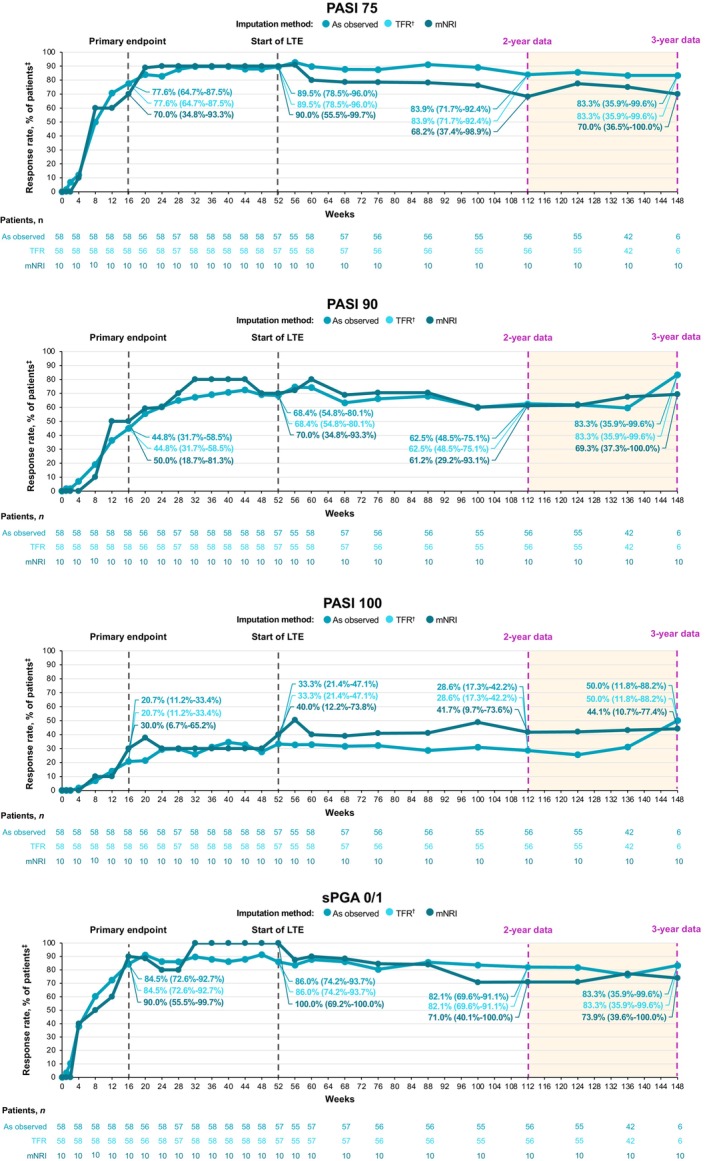
Efficacy is reported through 3 years in Japanese patients with plaque psoriasis in POETYK PSO‐4. ^†^Treatment failure rule (TFR) analysis captures discontinuations coded as “lack of efficacy” or “worsening of psoriasis.” ^‡^Modified nonresponder imputation (mNRI) analysis captures discontinuations coded as “worsening of psoriasis.” Data callouts represent the response rate (95% confidence interval). LTE, long‐term extension; mNRI, modified nonresponder imputation; PASI 75/90/100, ≥75%/≥90%/100% reduction from baseline in Psoriasis Area and Severity Index; sPGA 0/1, static Physician Global Assessment score of 0 (clear) or 1 (almost clear) with ≥2‐point improvement from baseline.

Deucravacitinib was effective through 3 years in Japanese patients with moderate to severe plaque psoriasis. In PSO‐1, high rates of clinical response achieved at 1 year (PASI 75, 88.9%; sPGA 0/1, 74.1%) were maintained through 3 years (PASI 75, 87.5%; sPGA 0/1, 66.7%; as observed) among patients receiving continuous deucravacitinib treatment from day 1 (*n* = 27; Figure 3). Among patients who crossed over from placebo to deucravacitinib at week 16 (*n* = 13), improvements in clinical response rates observed at 1 year (PASI 75, 92.3%; sPGA 0/1, 76.9%) were maintained through 3 years (PASI 75, 91.7%; sPGA 0/1, 91.7%) (Figure 4). PASI 90 and PASI 100 response rates at 1 year were generally consistent through 3 years in patients receiving continuous deucravacitinib treatment (PASI 90, 66.7% and 41.7%; PASI 100, 29.6% and 20.8%, respectively) and in placebo crossovers (PASI 90, 69.2% and 58.3%; PASI 100, 38.5% and 25.0%) (Figures 3 and 4). In PSO‐4, high response rates achieved at 1 year (PASI 75, 89.5%; PASI 90, 68.4%; PASI 100, 33.3%; sPGA 0/1, 86.0%) were also maintained through 3 years (PASI 75, 83.3%; PASI 90, 83.3%; PASI 100, 50.0%; sPGA 0/1, 83.3%) in patients with plaque psoriasis receiving continuous deucravacitinib treatment (*n* = 58) (Figure 5). Results were consistent using as‐observed values and the data imputation methodologies mNRI and TFR.

## 4 DISCUSSION

This analysis of Japanese patients from the phase 3 PSO‐1 trial and the phase 3 PSO‐4 trial who enrolled in the LTE trial demonstrates that long‐term deucravacitinib treatment has an acceptable safety and tolerability profile and is effective in these patients with chronic moderate to severe plaque psoriasis. The 3‐year safety and tolerability profile of deucravacitinib in Japanese patients in the LTE trial is consistent with the 1‐year safety profile in the Japanese subgroup in PSO‐1 and the 1‐year safety profile in PSO‐4, with no new or emerging safety signals specific to the Japanese population identified during long‐term deucravacitinib treatment.^19^, ^20^ Additionally, efficacy rates were maintained at high levels through 3 years in Japanese patients who received continuous deucravacitinib treatment from baseline and in those who crossed over from placebo at week 16, with results demonstrating consistency regardless of analysis methodology used (as‐observed, mNRI, or TFR). Safety and efficacy outcomes through 3 years in Japanese patients were also consistent with those previously reported in the 2‐ and 3‐year analyses of the pooled global population in the PSO‐1, PSO‐2, and LTE trials.^21^, ^22^

AEs in Japanese patients continued to be predominantly mild or moderate in severity through 3 years, and the incidence rates for serious AEs and AEs resulting in treatment discontinuation remained low. Similar to the 1‐year analyses of PSO‐1 and PSO‐4, and the 2‐ and 3‐year analyses of the global PSO‐1, PSO‐2, and LTE trials, nasopharyngitis was the most frequently reported AE.^17^, ^19^, ^20^, ^21^, ^22^ Previous studies have reported that JAK type 1, 2, and 3 inhibitors are associated with increased risks of serious infections, cardiovascular events, and other AEs of concern.^28^, ^29^ However, incidence rates of AEs of interest, including serious infections, malignancies, cardiovascular events, and skin events, were low with deucravacitinib treatment and comparable to rates reported at 1 year in PSO‐1 and PSO‐4, as well as to rates reported in the 2‐ and 3‐year analyses of the pooled global population.^17^, ^19^, ^20^, ^21^, ^22^ Herpes zoster events, an AE of interest with increased risk in Japanese patients, were localized and nonserious and did not require treatment discontinuation. The incidence rate for herpes zoster events in Japanese patients through 3 years (EAIR, 1.60/100 PYs) was somewhat higher than that reported through 3 years in the global population (EAIR, 0.60/100 PYs) and epidemiologic rates reported in the Japanese general population (EAIR, 0.65/100 PYs).^22^, ^30^ The incidence rate of herpes zoster events at 1 year was also higher in the POETYK PSO‐3 (NCT04167462) trial (2.7/100 PYs), another phase 3 trial that evaluated the efficacy and safety of deucravacitinib in East Asian patients with moderate to severe plaque psoriasis, than in PSO‐1 (1.2/100 PYs; included Asian patients) and in PSO‐2 (0.7/100 PYs; conducted in a non‐Asian population)^17^, ^18^, ^31^ For unclear reasons, herpes zoster incidence rates are also higher in East Asian populations than in populations from other parts of the world when exposed to agents that increase the risk of herpes zoster reactivation, such as JAK type 1, 2, and 3 inhibitors and type I interferon pathway inhibitors.^32^, ^33^, ^34^, ^35^, ^36^ However, in both the current analysis and in PSO‐3, the number of herpes zoster events was limited and, as a result, it cannot be definitively concluded that deucravacitinib treatment increases the risk of herpes zoster, although it is possible that TYK2 inhibition slightly increases the risk in an East Asian population compared with other populations. Additional real‐world safety data will be needed to clarify this issue further. Tuberculosis events and hepatitis B reactivation or new infection events were not reported in Japanese patients receiving deucravacitinib treatment through 3 years; however, appropriate screening and monitoring for these events are still important. Finally, evaluation of CPK, a laboratory parameter of interest, showed no clinically meaningful changes in levels through 3 years.

Deucravacitinib treatment demonstrated durable efficacy through 3 years in Japanese patients with moderate to severe plaque psoriasis. Clinical efficacy outcomes, including PASI 75 and sPGA 0/1 response rates, were maintained through 3 years in patients receiving continuous deucravacitinib treatment from baseline and were improved from week 16 among patients crossing over from placebo to deucravacitinib treatment. PASI 90 and PASI 100 response rates were generally consistent through 3 years, given the limited numbers of patients included in these analyses. Minor differences between response rates in PSO‐1 and PSO‐4 may be attributable to the small numbers of Japanese patients in each trial. Although PASI 90 response rates decreased, patients maintained PASI 75 response rates through 3 years. Since the mean baseline PASI score for patients in this analysis was 21.6, PASI 90 response rates indicate that these patients may retain an absolute PASI of approximately 3 to 5. Notably, approximately one‐fifth of patients achieved total clearance of psoriasis (PASI 100) through 3 years. Additional real‐world data are needed to further evaluate the long‐term efficacy of deucravacitinib treatment in Japanese patients with plaque psoriasis.

Study limitations include the relatively low number of Japanese patients with plaque psoriasis enrolled in PSO‐1 and in PSO‐4 who completed 3 years of deucravacitinib treatment. The PSO‐4 trial was initiated after the PSO‐1 trial; as a result, fewer patients in PSO‐4 compared with PSO‐1 had completed 3 years by the data cutoff date, limiting the number of PSO‐4 patients in this efficacy analysis. Additional limitations include the preponderance of male patients in this analysis (male to female ratio, 3:1); however, this distribution is consistent with the established prevalence of moderate to severe plaque psoriasis in Japan.^37^ Real‐world studies will provide additional insights about the benefits and risks associated with the long‐term use of deucravacitinib treatment in the Japanese patient population.

In conclusion, this analysis provides additional support that deucravacitinib, an oral, selective, allosteric TYK2 inhibitor, has an acceptable safety and tolerability profile and provides durable efficacy through 3 years in Japanese patients with plaque psoriasis.

## FUNDING INFORMATION

This study was sponsored by Bristol Myers Squibb.

## CONFLICT OF INTEREST STATEMENT

These clinical trials were sponsored by Bristol Myers Squibb. A.M. has received honorarium as meeting chair/lecturer from AbbVie, Boehringer Ingelheim Japan, Eisai, Eli Lilly Japan K.K., Janssen Pharmaceutical K.K., Kyowa Kirin, Maruho Co., Mitsubishi Tanabe Pharma, Novartis Pharma K.K., Taiho Pharmaceutical, Torii Pharmaceutical, and Ushio; funding from AbbVie GK, Eisai, Eli Lilly Japan K.K., Kyowa Hakko Kirin, Leo Pharma KK, Maruho, Mitsubishi Tanabe Pharma, Novartis Pharma K.K., Taiho Pharmaceutical, and Torii Pharmaceutical; and consulting fees from AbbVie GK, Boehringer Ingelheim Japan, Bristol Myers Squibb, Eli Lilly Japan K.K., Janssen Pharmaceutical K.K., Kyowa Kirin, Maruho, Mitsubishi Tanabe Pharma, Nippon Kayaku, Novartis Pharma K.K., NPO Health Institute Research of Skin, Sun Pharma, Torii Pharmaceutical, and UCB Japan. S.I. has received grants and personal fees from AbbVie, Eisai, Janssen, Kyowa Kirin, Leo Pharma, Maruho, Sun Pharma, Taiho Yakuhin, Tanabe Mitsubishi, and Torii Yakuhin and personal fees from Amgen (Celgene), Boehringer Ingelheim, Bristol Myers Squibb, Daiichi Sankyo, Lilly, Novartis, and UCB. Y.T. has received research grants from AbbVie, Amgen, Boehringer Ingelheim, Bristol Myers Squibb, Eisai, Jimro, Kyowa Kirin, Leo Pharma, Lilly, Maruho, Sun Pharma, Taiho Pharmaceutical, Tanabe‐Mitsubishi, Torii Pharmaceutical, and UCB and honoraria from AbbVie, Amgen, Boehringer Ingelheim, Bristol Myers Squibb, Eisai, Janssen, Jimro, Kyowa Kirin, Leo Pharma, Lilly, Maruho, Novartis, Pfizer, Sun Pharma, Taiho Pharmaceutical, Tanabe‐Mitsubishi, Torii Pharmaceutical, and UCB. Y.O. has received research grants from AbbVie, Eisai, Maruho, Shiseido, Sun Pharma, and Torii; honoraria from AbbVie, Amgen, Boehringer Ingelheim, Bristol Myers Squibb, Eisai, Janssen, Jimro, Kyowa Kirin, Leo Pharma, Lilly, Maruho, Novartis Pharma, Pfizer, Sanofi, Sun Pharma, Taiho Pharmaceutical, Tanabe‐Mitsubishi, Torii Pharmaceutical, and UCB; and has received funding for clinical trials from AbbVie, Amgen, Boehringer Ingelheim, Bristol Myers Squibb, Celgene, Janssen Pharma, Leo Pharma, Lilly, Maruho, Pfizer, Sun Pharma, and UCB. K.H. is an employee of Bristol Myers Squibb. R.M.K., and K.T. are employees of and shareholders in Bristol Myers Squibb. S.B. was an employee at the time of study conduct and stockholder: Bristol Myers Squibb. K.H. was a consultant to Bristol Myers Squibb via Syneos Health at the time of study conduct. M.O. has received honoraria and/or research grants from AbbVie, Amgen, Boehringer Ingelheim, Bristol Myers Squibb, Celgene, Eisai, Janssen, Kyowa Kirin, Leo Pharma, Lilly, Maruho, Mitsubishi Tanabe Pharma, Nichi‐Iko, Nippon Kayaku, Novartis, Pfizer, Sanofi, Sun Pharma, Taiho Pharmaceutical, Torii Pharmaceutical, and UCB. Shinichi Imafuku and Yayoi Tada are Editorial Board members of *Journal of Dermatology* and coauthors of this article. To minimize bias, they were excluded from all editorial decision‐making related to the acceptance of this article for publication.

## Supporting information


Data S1.

## Data Availability

Bristol Myers Squibb describes their data‐sharing request process on the following website: https://www.bms.com/researchers‐and‐partners/independent‐research/data‐sharing‐request‐process.html.
