# Supplementary material for: Deucravacitinib in plaque psoriasis: Safety and efficacy through 3 years in Japanese patients in the phase 3 POETYK PSO‐1, PSO‐4, and LTE trials
Source: J Dermatol. 2025 Mar 11;52(5):761–72. doi: 10.1111/1346-8138.17685 (PMC12056280; doi:10.1111/1346-8138.17685)

**SUPPORTING INFORMATION**

**Deucravacitinib in plaque psoriasis: Safety and efficacy through 3 years in Japanese patients in the phase 3 POETYK PSO-1, PSO-4, and LTE trials**

Akimichi Morita, Shinichi Imafuku, Yayoi Tada, Yukari Okubo, Katsuyoshi Habiro, Katsuki Tsuritani, Subhashis Banerjee, Kim Hoyt, Renata M. Kisa, Mamitaro Ohtsuki

**TABLE S1.** Serious adverse events in Japanese patients with plaque psoriasis in the pooled POETYK PSO-1, PSO-4, and LTE population.

| **Serious AE category** | **Deucravacitinib^†^ (*n* =125)**  **Total exposure, PY = 322.7** | |
| --- | --- | --- |
|  | ***n* (%)** | **EAIR/100 PY**  **(95% CI)** |
| Serious AE | 21 (16.8) | 7.4 (4.8, 11.4) |
| Unstable angina | 2 (1.6) | 0.6 (0.2, 2.6) |
| Acute myocardial infarction | 1 (0.8) | 0.3 (0.0, 2.3) |
| Acute promyelocytic leukemia^‡^ | 1 (0.8) | 0.3 (0.0, 2.3) |
| Asthma | 1 (0.8) | 0.3 (0.0, 2.3) |
| Alcoholic ketoacidosis | 1 (0.8) | 0.3 (0.0, 2.3) |
| Angina pectoris^‡^ | 1 (0.8) | 0.3 (0.0, 2.3) |
| Calculus, urinary | 1 (0.8) | 0.3 (0.0, 2.3) |
| Cataract | 1 (0.8) | 0.3 (0.0, 2.3) |
| Cerebral infarction | 1 (0.8) | 0.3 (0.0, 2.3) |
| Chronic inflammatory demyelinating polyradiculoneuropathy | 1 (0.8) | 0.3 (0.0, 2.3) |
| Clavicle fracture | 1 (0.8) | 0.3 (0.0, 2.3) |
| Colorectal cancer | 1 (0.8) | 0.3 (0.0, 2.3) |
| Coronavirus infection^‡^ | 1 (0.8) | 0.3 (0.0, 2.3) |
| COVID-19 | 1 (0.8) | 0.3 (0.0, 2.3) |
| Dyspnea, exertional | 1 (0.8) | 0.3 (0.0, 2.3) |
| Extraskeletal ossification | 1 (0.8) | 0.3 (0.0, 2.3) |
| Gastric cancer^‡^ | 1 (0.8) | 0.3 (0.0, 2.3) |
| Large intestine polyp | 1 (0.8) | 0.3 (0.0, 2.3) |
| Normal pressure hydrocephalus | 1 (0.8) | 0.3 (0.0, 2.3) |
| Osteoarthritis | 1 (0.8) | 0.3 (0.0, 2.3) |
| Pneumonia^‡^ | 1 (0.8) | 0.3 (0.0, 2.3) |
| Pyelonephritis | 1 (0.8) | 0.3 (0.0, 2.3) |
| Pyrexia | 1 (0.8) | 0.3 (0.0, 2.3) |

Abbreviations: CI, confidence interval; EAIR, exposure-adjusted incidence rate; PY, person-years.
^†^Pooled Japanese patients from the parent trials (POETYK PSO-1 and PSO-4) and the POETYK LTE who received ≥ 1 dose of deucravacitinib through the data cutoff date of June 15, 2022.
^‡^Treatment-related per investigator.

**Figure S1.** Patient disposition as of June 15, 2022. ^†^This represents the population of patients who received deucravacitinib in a parent trial (POETYK PSO-1 or PSO-4) and/or the POETYK LTE trial through the data cutoff date of June 15, 2022. Given the study designs, some patients may have received placebo and deucravacitinib, or apremilast and deucravacitinib during the first year in POETYK PSO-1.


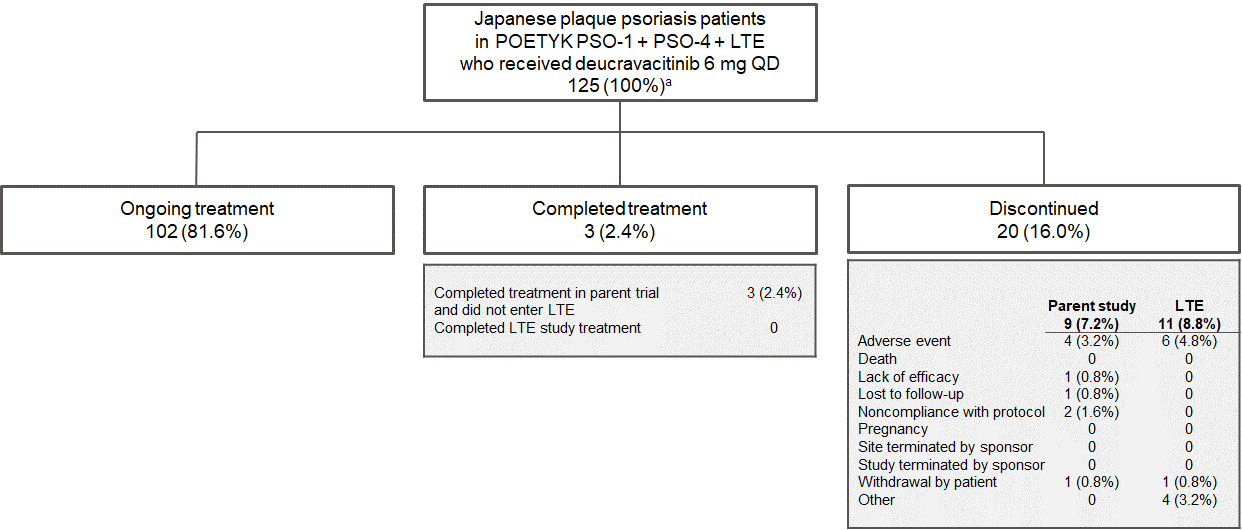

Supplement: Supplementary file 1 — Data S1. [file JDE-52-761-s001.docx]
